# Infant survival in western lowland gorillas after voluntary dispersal by pregnant females

**DOI:** 10.1007/s10329-020-00844-z

**Published:** 2020-07-27

**Authors:** Marie L. Manguette, Thomas Breuer, Jana Robeyst, Vidrige H. Kandza, Martha M. Robbins

**Affiliations:** 1 Department of Primatology, Max Planck Institute for Evolutionary Anthropology, Deutscher Platz 6, 04103 Leipzig, Germany; 2 Mbeli Bai Study, Wildlife Conservation Society Congo Program, B.P. 14537, Brazzaville, Congo; 3 World Wide Fund for Nature, Reinhardtstrasse 18, 10117 Berlin, Germany

**Keywords:** *Gorilla gorilla*, Female secondary dispersal, Pregnant female transfer, Infanticide risk, Female mate choice

## Abstract

In many social species, after the alpha male has been replaced or the group disintegrates, a female’s infant is at risk of infanticide by a male. Female gorillas have developed the rare strategy of secondary dispersal in which they transfer between reproductive groups during the limited time period between weaning an infant and conceiving the next one (voluntary dispersal). By doing so they leave a weaker silverback near the end of his tenure and join a stronger silverback at an earlier stage of his own tenure, thereby mitigating the risk of infanticide if the former dies. If females are pregnant or have unweaned offspring when the only male in the group dies, their offspring are vulnerable to infanticide by the new silverback that they join (via involuntary dispersal). In the few known cases of female gorillas transferring when pregnant (mainly after group disintegration), their offspring were killed. We report here on three adult females that transferred voluntarily while pregnant multiple times between two groups yet their offspring were not killed by the new group’s silverback. The gorillas were observed from 1995 to 2015 at the Mbeli Bai research site in northern Republic of the Congo. The females gave birth 5–6 months (gestation period 8.5 months) after their last transfer. To our knowledge, these observations are the first to show that wild female western lowland gorillas can transfer voluntarily while pregnant without incurring infanticide by a new silverback. These observations highlight the behavioural plasticity shown by female gorillas in response to sexual coercion by males.

**Electronic supplementary material:**

The online version of this article (10.1007/s10329-020-00844-z) contains supplementary material, which is available to authorized users.

## Introduction

Social mammals exhibit a range of intersexual conflicts and compromises (Clutton-Brock 2007). One clear example of sexual conflict suffered by reproductive females is infanticide by males (Lukas and Huchard 2014), which has been observed in a wide range of mammals, e.g. in rodents, ungulates, carnivores, pinnipeds and primates (van Schaik 2000a). Infanticide reduces the reproductive success of both parents, but it can increase the reproductive success of the infanticidal male if three conditions are met: there is a low probability that the infanticidal male sired the infant, the mother resumes reproduction sooner if the infant is killed, and the mother is likely to mate with the infanticidal male (Hrdy 1977; Smuts and Smuts 1993).

Female primates have developed several strategies to avoid infanticide, such as forming permanent associations with males that provide protection against other males, remaining with the father until weaning the infant, residing in a multi-male group, mating with multiple males to create paternity confusion, or joining a better protector male using secondary dispersal (Palombit 2015). Secondary dispersal (also referred to as transfer), which involves the movement of females between reproductive groups, is found in only a few mammals [e.g. Thomas langurs (Sterck et al. 2005); tropical bats (Nagy et al. 2007); feral horses (Debeffe et al. 2015); gorillas (Stokes et al. 2003; Robbins and Robbins 2015)] and is believed to represent female choice for a high-quality male where it occurs voluntarily (Harcourt and Stewart 2007; Lukas and Huchard 2014). However, it may also occur involuntarily after group disintegration [in gorillas (Stokes et al 2003)] or female eviction [in ursine colobus (Teichroeb et al. 2009)] and lead to infanticide if the females are pregnant or have unweaned offspring. When secondary dispersal occurs voluntarily, this ability to move between groups gives females an advantage over those of philopatric species as it allows them to reduce the risk of infanticide of their young (Lewis 2018). However, when females disperse, the timing of their voluntary dispersal must be carefully calculated, as due to the risk of infanticide, they have only a very short window of time in which to leave their current group between weaning an infant and conceiving again (Sterck et al. 2005; Robbins and Robbins 2015; Sicotte 1993; Sicotte et al. 2017). If a female transfers while pregnant or with an infant, the first and third criteria for infanticide are met, and the infant is at risk.

Infanticide by males has been directly observed or inferred in many populations of gorillas (Watts 1989; Yamagiwa et al. 2009; Breuer et al. 2010; Robbins et al. 2013). Infanticide is often inferred after the silverback’s death and subsequent group disintegration when infants disappear soon after their mother’s transfer to a new silverback (Robbins et al. 2013). However, cases of unweaned infants surviving after transferring together with their mother have been observed (Sicotte 2000; Stokes et al. 2003). The infanticide rate after death of the silverback (leading to group disintegration) is quite high (12%) in western lowland gorillas at Mbeli (Breuer et al. 2010; Robbins et al. 2013). With their strategy of voluntary secondary dispersal, female gorillas may be able to choose a better male before the death of the current silverback and protect their offspring from infanticide (Palombit 2015; Manguette et al. 2019, 2020).

In gorillas, dispersal of females while they are pregnant is known to occur after group disintegration, when females are forced to find another male since they typically do not range on their own. Transfer of females while pregnant after involuntary dispersal has been reported both at Mbeli Bai and at Kahuzi-Biega, and in all instances the infants were killed soon after birth (Stokes et al. 2003; Yamagiwa et al. 2009). Thus, the voluntary transfer of a female while pregnant carries a very high risk of infanticide. Here we report three cases of voluntary transfer by pregnant wild western lowland gorillas that did not result in the death of their young.

## Methods

### Study site and data collection

Western gorillas were studied at Mbeli Bai, a swampy forest clearing in the Nouabale-Ndoki National Park, northern Republic of the Congo. We observed the gorillas from a 9-m-high observation platform overlooking the bai with almost 100% visibility [for further details, see Parnell (2002) and Breuer et al. (2009)]. The Mbeli population demographic dataset comprised 440 individually identifiable gorillas (including 100 adult females in 40 different breeding groups) observed from February 1995 to November 2015. We specifically discuss three females (Ndebele, Khoisan and Efi) and four different groups (Gretsky, Boris, Zulu and Saha). The groups were observed on average between 19 and 38 times a year at the clearing (Table 1). Gretsky and Zulu, the group of origin of the females, were composed of 14 and 12 individuals, respectively, while Boris and Saha, the silverbacks the females transferred to, were both solitary (Table 1).

**Table 1:** Summary of the study groups used for this study

Group name	Years observed^a^	Visits per year^b^	Group composition^c^					
			SB	AF	BB	SA	Juveniles	Infants
Gretsky	2004–2015	30	1	7		2	2	2
Boris	2014–2015	19	1					
Zulu	2000–2015	36	1	4	1	1	4	1
Saha	2013–2015	38	1					

*SB* Silverbacks, *AF* adult females, *BB* blackbacks, *SA* male and female subadults [see Breuer et al. (2009) for a description of the classification]

^a^First and last years of observation of a reproductive group

^b^Average number of visits to the clearing as a reproductive group or as a solitary silverback per year of observation

^c^Before conception by the transferring females

Due to the nature of observations at the bai, not all of the gorillas were observed daily and dates of birth and dispersal were estimated. Some infants were observed within a few days of their birth, as confirmed by previous recent observations of the group without them. Beyond a few days of age, birthdates were estimated by comparing the infants’ morphological and behavioural characteristics with those of infants of known ages (Parnell 2002; Nowell and Fletcher 2007; Breuer et al. 2009). The earliest possible birthdate was assumed to be the last date on which the mother was observed without her infant. The latest possible birthdate was assumed to be the first date that the mother was observed with her infant. Gorillas have an average gestation period of 255 days with a range of 240–280 days (Harcourt and Stewart 2007; Phillips et al. 2015). The expected conception date equalled the estimated birthdate minus 255 days. The earliest possible conception date equalled the earliest possible birthdate minus 280 days. The latest possible conception date equalled the latest possible birthdate minus 240 days (Table S1).

Dispersal dates were estimated as the midpoint between the last visit to the bai of the group of origin and the first visit of the group of destination (Manguette et al. 2019). We accounted for the maximum possible error by assuming that a female transferred and conceived on the day she was last observed in the previous group and that she gave birth on the first day she was observed with her offspring in the new group (Table S2).

## Results

**Fig. 1:**
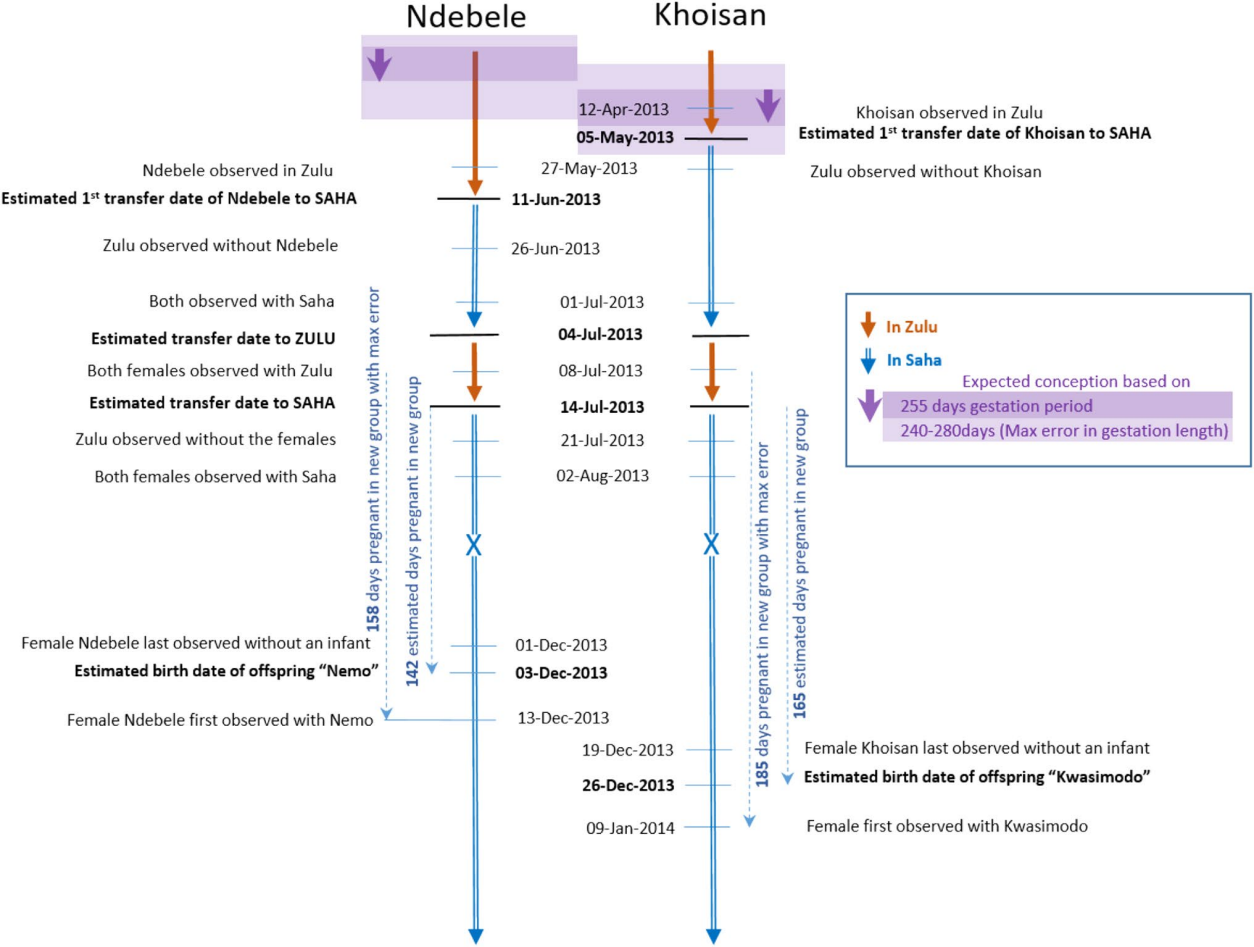
Details of the dispersal of the two females, Ndebele, Khoisan, who transferred while pregnant. To calculate the maximum error, we assumed that each female conceived on the day she was last observed in the previous group (if she transferred on that day) and gave birth on the day she was first observed with her infant in the new group

We report the dispersal of three females (Ndebele, Khoisan, and Efi) that transferred multiple times during pregnancy (Table S3 for all observation dates). Ndebele had been a member of Zulu’s group (Zulu was estimated to be 33 years old) for 13 years when she conceived between February and April 2013 (Fig. 1). She transferred to a solitary male, Saha (estimated to be 24 years old), between May and June, then briefly resided in Zulu’s group for a few weeks again in July, then returned to Saha’s group in August where she remained. She had been with Saha for the last 142 days of her gestation period when her next offspring (Nemo) was born in December 2013.

**Fig. 2:**
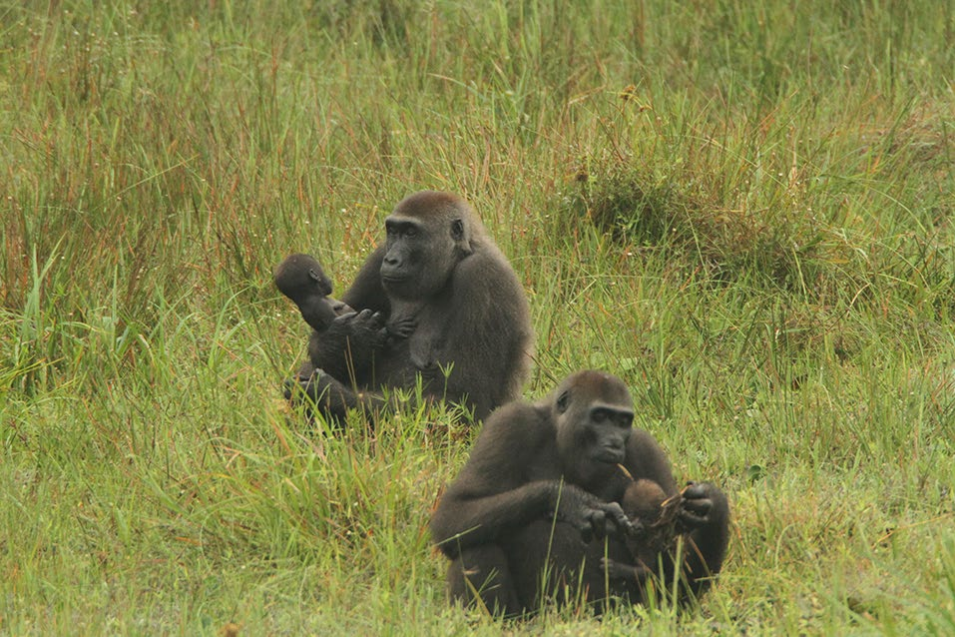
Photo of Ndebele with her infant Nemo (*left*) and Khoisan with her infant Kwasimodo (*right*) in January 2014

Khoisan had also been observed in Zulu’s group for 13 years when she conceived between March and May 2013 (Fig. 1). Her range of potential conception dates overlapped with her initial transfer to Saha between April and May. She briefly visited Zulu’s group again in July, then returned to Saha’s group where she remained from August onwards. She had been with Saha for the last 165 days of her gestation period when her next offspring (Kwasimodo) was born in December. The two offspring born in Saha’s group were both healthy (no sign of preterm birth; Fig. 2), which suggests that the gestation period was normal in both cases.

**Fig. 3:**
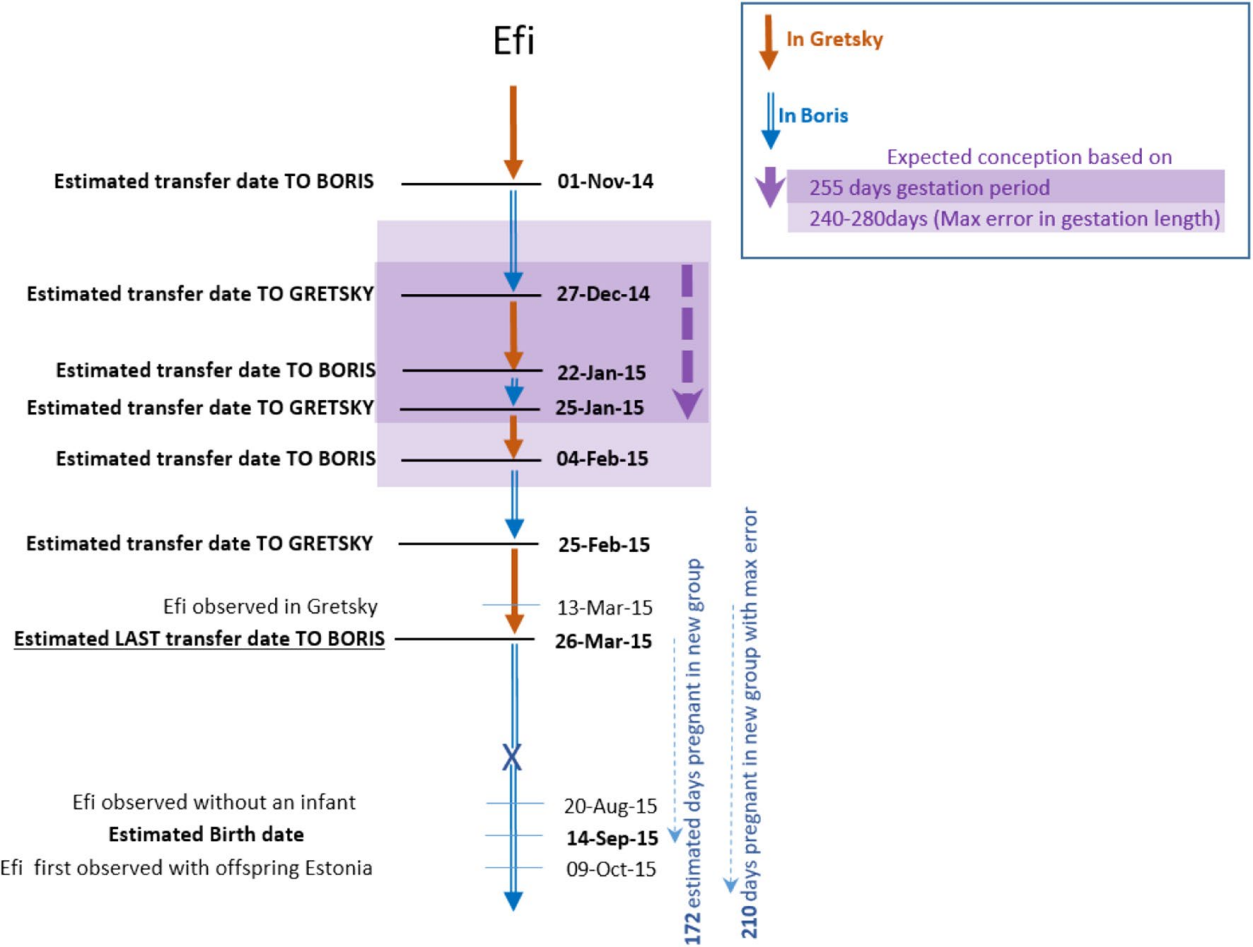
Details of the dispersal of the female, Efi, who transferred multiple times before and after conceiving. Both Gretsky and Boris were likely fathers of Efi’s offspring, Estonia. The dates used to estimate the conception dates and error are given in Table S1, and the dates used to calculate the estimated transfer dates are given in Table S2

From November 2014 up to and including March 2015, Efi transferred seven times between the group of Gretsky (whose estimated age was 30 years) and a solitary male, Boris (whose estimated age was 24 years). She resided with each male during her range of potential conception dates between November 2014 and February 2015 (Fig. 3). She had been with Boris for the last 172 days of her gestation period when her next offspring (Estonia) was born between August and October 2015.

In all three of these cases of dispersal by pregnant females, the infants survived with their mother in the new group until the end of the study (i.e. when the infants were 24, 23 and 2.5 months of age). These three births represent only 1.5% of the 202 births that occurred during the study, which suggests that dispersal by pregnant females is extremely rare.

## Discussion

Our findings show that in western lowland gorillas, although rare with regards to total births recorded in the current study (~1.5%), offspring born to mothers that voluntary transfer when pregnant may not be killed. All three females transferred multiple times between two different groups during their pregnancy, and potentially also during the period of conception of their offspring. However, as this strategy may result in infanticide, the question arises: why were these infants not killed by the new silverbacks?

It is likely that the new silverbacks did not kill the infants because they had some degree of paternity certainty or were actually the fathers. In the cases of Khoisan and Efi, the conception period may easily have coincided with a time when these females were with the new silverbacks during earlier transfers (Figs. 1, 2). Even in the case of Ndebele, we may have missed a previous transfer during the conception period if it lasted for only a few days (Table S3). Furthermore, the transferring female may have confused the new silverback by mating with him soon after transfer, as it is known that female gorillas copulate throughout pregnancy (Robbins 2003; Doran‐Sheehy et al. 2009). (Copulation by these three females was not observed, but this is not surprising given that copulation could only be rarely observed due to the presence of swampy vegetation in the clearing.) The mating behaviour of a transferring female may thus play a decisive role in preventing infanticide (van Schaik 2000b). However, the effectiveness of mating to confuse paternity and prevent infanticide decreases as pregnancy progresses. For example, infanticidal attacks after group takeovers were prevented when females mated during pregnancy until around 80 days before parturition in red colobus (151-day gestation period), 60 days in Hanuman langurs (198-day gestation period), 45 days in blue monkeys (138-day gestation period) and 40 days in sooty mangabeys (165-day gestation period) (van Schaik 2000b). Thus females in these species seem to be able to successfully prevent infanticide by spending at least 25–50% of their gestation period with a new male. The Mbeli females spent > 50% of their gestation period with the new silverback. The only observed case of unsuccessful transfer of a pregnant female at Mbeli (after group disintegration) occurred when she spent only 2 months with the new silverback (~ 25% of the gestation period) (Stokes et al. 2003). A prolonged period spent with the same male between the transfer (and potentially also the first mating) of a pregnant female and birth of her offspring may therefore be necessary to prevent infanticide. The timing of transfer may be more important than whether the dispersal is voluntary.

Another question remains as to why these females transferred while pregnant despite the substantial risk of infanticide. Hypothetically, a female may struggle to transfer during the limited time window between weaning her offspring and next conception if a silverback prevents her from doing so through herding (Sicotte 1993; Breuer et al. 2016). It is also possible that silverbacks of a group may avoid intergroup encounters to prevent a female from transferring (Watts 1994), which may occur more commonly in silverbacks that are older and weaker towards the end of their tenure (M. L. M., personal observation), or that a silverback’s physical condition may deteriorate suddenly after a female conceives. In these scenarios, poor male condition may lead to the transfer of females when they are pregnant, although a male’s condition rarely deteriorates suddenly after a female conceives. With regard to intergroup encounters, resident males typically cannot prevent these entirely. If a resident silverback is unable to protect offspring until weaning, then the lifetime reproductive success of a female might actually be higher if her infant is killed at birth (rather than postponing the inevitable). By deliberately risking infanticide, the dispersal of a pregnant female may be comparable to the termination of pregnancy observed in geladas (Roberts et al. 2012), or the abandonment of infants as seen in colobine monkeys (Sicotte et al. 2007).

Another possibility is that multiple transfers between two males is a bet-hedging strategy if a female is unsure which of the two is the better mate choice (Sicotte 2001). Females may need time with a new male/group to determine whether he/they are a better match than their current silverback (e.g. in terms of male quality, tolerance, mating ability, group size, and reaction of other females). In this regard, transferring multiple times between two males is somewhat analogous to male incursions or forays [e.g. in colobus monkeys (Sicotte and MacIntosh 2004)] and extra-group copulations seen in many mammalian species (Van Noordwijk and van Schaik 2000; Wolff and Macdonald 2004). In gorillas, extra-group copulations during inter-group encounters have been observed very rarely, and where they were observed involved immature individuals (Sicotte 2001). There is currently no evidence that infants are conceived during extra-group copulations in gorillas, e.g. a genetic study of 79 mountain gorilla infants that found that they were all sired in their respective groups (Bradley et al. 2005; Vigilant et al. 2015).

It is interesting that the extra-group paternity in our study population, where 1.5% of the infants lived in a group with a silverback that was not their father, is similar to that of human infants (1–2%) reported in a study where paternity confidence was high (Anderson 2006; Greeff and Erasmus 2015). Extra-group paternity, which is not uncommon in primates, is especially widespread in certain species (fat-tailed dwarf lemur, 44%; rhesus macaques, 36%; Japanese macaques, 33%), less common in others (chimpanzees, 7%; eastern lesser bamboo lemur, 8.5%) and completely absent in mountain gorillas and red-fronted lemurs (Isvaran and Clutton-Brock 2007). Isvaran and Clutton-Brock (2007) found that the number of cases of extra-group paternity increased in species with a short mating season and a large number of females where it was difficult for males to monopolize their mates. The low level of extra-group paternity found in our study of western lowland gorillas along with their lack of mating season and relatively small groups agrees with these findings. The low level of extra-group paternity may explain why males invested in the care of these infants even when there was doubt surrounding their paternity.

Our results suggest that pregnant females in the western lowland gorilla may be able to transfer to a new group under the right conditions (less than halfway into the gestation period) without incurring infanticide by a male, and highlight the behavioural plasticity that these females show in response to sexual coercion by males. The cases highlighted here do not challenge the hypothesis of sexually selected infanticide, as not every infant needs to be killed by a non-parent male to support this evolutionary strategy (van Schaik 2000b).

## Electronic supplementary material

Below is the link to the electronic supplementary material.

Supplementary file1 (DOCX 38 kb)
