# Supplementary material for: Infant survival in western lowland gorillas after voluntary dispersal by pregnant females
Source: Primates. 2020 Jul 27;61(6):743–9. doi: 10.1007/s10329-020-00844-z (PMC7599133; doi:10.1007/s10329-020-00844-z)
Supplement: Supplementary file 1 — Supplementary file1 (DOCX 38 kb) [file 10329_2020_844_MOESM1_ESM.docx]

**Supplementary information**

**Infant survival in western lowland gorillas after voluntary dispersal by pregnant female**

Journal: Primates

Marie L. Manguette, Thomas Breuer, Jana Robeyst, Vidrige H. Kandza & Martha M. Robbins

***Corresponding author:***

**Marie L. Manguette**

Max Planck Institute for Evolutionary Anthropology, Deutscher Platz 6, 04103 Leipzig, Germany

Email: [marie_manguette@eva.mpg.de](mailto:marie_manguette@eva.mpg.de)

**SUPPLEMENTS**

**Additional information on the study groups**

In addition to the transfers of Ndebele and Khoisan, Zulu had two other females transfer out of his group in 2013, suggesting that he may have been getting old (and losing competitive ability), although he retained one female with an infant at the end of the study. Saha, on the other hand, had three females transfer into his group in 2013-2014, and by the end of the study period four infants had been born in his group. Gretsky had six adult females in his group at the end of the study, and Boris was not joined by any other females than Efi.

**Table S1:** Estimated birthdates and conception dates for three females who transferred while pregnant (See Methods for calculations of the earliest possible dates, the expected dates, and the latest possible dates).

| **Name of mother** | **Earliest birthdate of offspring** | **Estimated**  **birthdate of offspring** | **Latest birthdate of offspring** | **Earliest conception** | **Expected conception** | **Latest conception** |
| --- | --- | --- | --- | --- | --- | --- |
| **Khoisan** | 19-Dec-13 | 26-Dec-13 | 9-Jan-14 | 14-Mar-13 | 15-Apr-13 | 14-May-13 |
| **Ndebele** | 1-Dec-13 | 3-Dec-13 | 13-Dec-13 | 24-Feb-13 | 23-Mar-13 | 17-Apr-13 |
| **Efi** | 20-Aug-15 | 14-Sep-15 | 9-Oct-15 | 13-Nov-14 | 2-Jan-15 | 11-Feb-15 |

**Table S2:** demographic transfer data. G1name is the group the female transferred from. G2name is the group the female transferred into. G1before is the last date the female was observed in the current group, g1after is the first date the group was observed without the female, g2before is the last time the next group was observed without the female and g2after is the first time the group was observed with the female.

| **Female name** | **g1name** | **g2name** | **expected transfer date** | **g1before** | **g1after** | **g2before** | **g2after** |
| --- | --- | --- | --- | --- | --- | --- | --- |
| Khoisan | Zulu | Saha | 05-May-13 | 12-Apr-13 | 27-May-13 | 14-Apr-13 | 01-Jul-13 |
| Khoisan | Saha | Zulu | 04-Jul-13 | 01-Jul-13 | - | 27-Jun-13 | 08-Jul-13 |
| Khoisan | Zulu | Saha | 14-Jul-13 | 08-Jul-13 | 21-Jul-13 | - | 02-Aug-13 |
| Ndebele | Zulu | Saha | 11-Jun-13 | 27-May-13 | 26-Jun-13 | 14-Apr-13 | 01-Jul-13 |
| Ndebele | Saha | Zulu | 04-Jul-13 | 01-Jul-13 | - | 27-Jun-13 | 08-Jul-13 |
| Ndebele | Zulu | Saha | 14-Jul-13 | 08-Jul-13 | 21-Jul-13 | - | 2-Aug-13 |
| Efi | Gretsky | Boris | 01-Nov-14 | 31-Oct-14 | 03-Nov-14 | 13-Jun-14 | 10-Nov-14 |
| Efi | Boris | Gretsky | 27-Dec-14 | 10-Nov-14 | 08-Jan-15 | 15-Dec-14 | 04-Jan-15 |
| Efi | Gretsky | Boris | 22-Jan-15 | 04-Jan-15 | 23-Jan-15 | 21-Jan-15 | 22-Jan-15 |
| Efi | Boris | Gretsky | 25-Jan-15 | 22-Jan-15 | 27-Jan-15 | 23-Jan-15 | 28-Jan-15 |
| Efi | Gretsky | Boris | 04-Feb-15 | 31-Jan-15 | 13-Feb-15 | 27-Jan-15 | 08-Feb-15 |
| Efi | Boris | Gretsky | 25-Feb-15 | 24-Feb-15 | 27-Feb-15 | 15-Feb-15 | 13-Mar-15 |
| Efi | Gretsky | Boris | 26-Mar-15 | 13-Mar-15 | 08-Apr-15 | 27-Feb-15 | 01-Jun-15 |

Table S3: Observation days for each group/females starting 1 month prior to the estimated conception date of the female (Khoisan=15-Apr-13, Ndebele=23-Mar-13, Efi=2-Jan-15) and 1 month after the last transfer (Khoisan & Ndebele=14-July-13, Efi=26-Mar-15). We added a few months before and after for Efi to all the transfer periods.

| Date | Group name | Tot number gorillas present | Female Khoisan present | Female Ndebele  Present | Time group observed^1^ in minutes | Comments |
| --- | --- | --- | --- | --- | --- | --- |
| 22-Mar-13 | Zulu | 12 | Yes | Yes | 183 |  |
| 23-Mar-13 | Saha | 1 | No | No | 160 |  |
| 23-Mar-13 | Zulu | 12 | Yes | Yes | 161 |  |
| 24-Mar-13 | Zulu | 1 | No | Yes | 33 | Only Ndebele present |
| 26-Mar-13 | Zulu | 12 | Yes | Yes | 91 |  |
| 27-Mar-13 | Zulu | 12 | Yes | Yes | 151 |  |
| 28-Mar-13 | Saha | 1 | No | No | 62 |  |
| 31-Mar-13 | Saha | 1 | No | No | 99 |  |
| 01-Apr-13 | Zulu | 12 | Yes | Yes | 119 |  |
| 07-Apr-13 | Saha | 1 | No | No | 139 |  |
| 12-Apr-13 | Zulu | 12 | Yes | Yes | 67 |  |
| 14-Apr-13 | Saha | 1 | No | No | 128 |  |
| 27-May-13 | Zulu | 10 | No | Yes | 209 | Zulu observed without Khoisan |
| 26-Jun-13 | Zulu | 9 | No | No | 111 | Zulu observed without Ndebele |
| 27-Jun-13 | Zulu | 4 | No | No | 162 |  |
| 01-Jul-13 | Saha | 3 | Yes | Yes | 75 | Both females observed in Saha |
| 08-Jul-13 | Zulu | 11 | Yes | Yes | 237 | Both females observed in Zulu |
| 21-Jul-13 | Zulu | 8 | No | No | 47 | Zulu without both females |
| 22-Jul-13 | Zulu | 9 | No | No | 195 |  |
| 02-Aug-13 | Saha | 3 | Yes | Yes | 137 | Both females observed in Saha |
| 06-Aug-13 | Zulu | 9 | No | No | 191 |  |
| 07-Aug-13 | Zulu | 9 | No | No | 167 |  |
| 09-Aug-13 | Saha | 3 | Yes | Yes | 89 |  |
| 14-Aug-13 | Zulu | 9 | No | No | 63 |  |
|  |  |  | Female Efi  present | |  |  |
| 31-Oct-14 | Gretsky | 15 | Yes | | 134 |  |
| 03-Nov-14 | Gretsky | 4 | No* | | 90 | Only 4 individuals present |
| 10-Nov-14 | Boris | 2 | Yes | | 81 |  |
| 13-Nov-14 | Gretsky | 1 | No* | | 30 | Only the silverback of the group |
| 23-Nov-14 | Boris | 1 | No | | 75 |  |
| 27-Nov-14 | Boris | 1 | No | | 202 |  |
| 28-Nov-14 | Boris | 1 | Yes | | 35 | Efi alone but enters in the clearing through Boris’s area |
| 30-Nov-14 | Boris | 1 | No | | 154 |  |
| 03-Dec-14 | Gretsky | 3 | No* | | 191 | Only 3 individuals visited the bai (at this time Efi is considered with Boris) |
| 11-Dec-14 | Gretsky | 13 | No | | 162 |  |
| 15-Dec-14 | Gretsky | 14 | No | | 152 |  |
| 25-Dec-14 | Boris/ Gretsky | 1 | Yes | | 44 | Only Efi present, unknown group |
| 01-Jan-15 | Boris/ Gretsky | 1 | Yes | | 174 | Only Efi present, unknown group |
| 04-Jan-15 | Gretsky | 15 | Yes | | 159 |  |
| 08-Jan-15 | Boris | 1 | No | | 101 |  |
| 11-Jan-15 | Gretsky | 4 | No* | | 112 | Only 4 indivdiuals visited the bai |
| 18-Jan-15 | Boris | 1 | No | | 113 |  |
| 21-Jan-15 | Boris | 1 | No | | 126 |  |
| 22-Jan-15 | Gretsky | 14 | No | | 151 |  |
| 22-Jan-15 | Boris | 2 | Yes | | 79 |  |
| 23-Jan-15 | Gretsky | 1 | No* | | 29 | Only 1 female seen alone |
| 27-Jan-15 | Boris | 1 | No | | 21 |  |
| 28-Jan-15 | Gretsky | 15 | Yes | | 172 |  |
| 31-Jan-15 | Gretsky | 14 | Yes | | 180 |  |
| 08-Feb-15 | Boris | 2 | Yes | | 225 |  |
| 13-Feb-15 | Gretsky | 14 | No | | 160 |  |
| 14-Feb-15 | Boris | 1 | No | | 116 |  |
| 15-Feb-15 | Gretsky | 14 | No | | 141 |  |
| 17-Feb-15 | Boris | 1 | No | | 2 |  |
| 24-Feb-15 | Boris | 2 | Yes | | 111 |  |
| 27-Feb-15 | Boris | 1 | No | | 8 |  |
| 08-Mar-15 | Gretsky | 14 | No | | 111 |  |
| 09-Mar-15 | Gretsky | 14 | No | | 106 |  |
| 13-Mar-15 | Gretsky | 15 | Yes | | 140 |  |
| 08-Apr-15 | Gretsky | 14 | No | | 57 |  |
| 26-May-15 | Gretsky | 14 | No | |  |  |
| 1-Jun-15 | Gretsky | 3 | No | |  |  |
| 1-Jun-15 | Boris | 2 | Yes | |  | From this date on, female Efi is no longer observed with Grestky |

^1^from first individual entry to last individual leave *only a few individuals observed from the group, visit not used.
